# NXP-2 Positive Dermatomyositis: A Unique Clinical Presentation

**DOI:** 10.1155/2017/4817275

**Published:** 2017-06-13

**Authors:** Zeeshan Butt, Leeza Patel, Manash K. Das, Christopher A. Mecoli, Alim Ramji

**Affiliations:** ^1^Internal Medicine Residency Program, Prince George's Hospital Center, 3001 Hospital Dr, Cheverly, MD 20785, USA; ^2^Department of Rheumatology, University of Arkansas for Medical Sciences, Little Rock, AR, USA; ^3^Division of Rheumatology, John Hopkins University School of Medicine, 733 N Broadway, Baltimore, MD 21205, USA

## Abstract

Dermatomyositis (DM), a myopathy associated with inflammation and muscle weakness, has historically been difficult to diagnose. Recently, nuclear matrix protein (NXP-2) antibodies have been described as a myositis-specific antibody that may aid in the diagnostic evaluation. We present the case of a 21-year-old, previously healthy, African American male with DM. He presented to our outpatient clinic with periorbital swelling and a rash, for which he was started on prednisone by an ophthalmologist. Towards the end of the prednisone taper, he began to experience muscle weakness, a worsening rash, and dysphagia to solids with a resultant loss of 60 pounds within a month. He was transferred to a tertiary care hospital where he was further evaluated and ultimately diagnosed with dermatomyositis, supported by skin and muscle biopsies, and was found to be positive for NXP-2. He was given intravenous immunoglobulin (IVIG) and high-dose steroids with improvement.

## 1. Introduction

Dermatomyositis (DM) is a systemic disease characterized by chronic inflammation of the skin and muscle [1, 2]. There is significant clinical heterogeneity with respect to lung, muscle, joint, and cutaneous involvement, as well as variability in its association with malignancy and response to therapy [2–5]. Many patients with DM have circulating antibodies which are often associated with distinct clinical phenotypes [6, 7].

Recent studies identified new autoantibody specificities that include melanoma differentiation-associated protein 5 (MDA-5), transcription intermediary factor 1*γ* (TIF-1*γ*), and nuclear matrix protein (NXP-2) [8]. NXP-2 (also known as anti-MJ), a myositis-specific antibody, has been previously identified in 25% of juvenile dermatomyositis patients, and studies have shown its association with calcinosis and severe muscle weakness as well as potential gastrointestinal involvement [4, 7].

Here we present a case of adult NXP-2 positive DM.

## 2. Case Presentation

A 21-year-old African American male with no past medical history was in his usual state of health until January 2014, when he developed unusual periorbital swelling and a rash. He was evaluated at an outpatient ophthalmology clinic and was started on 60 mg prednisone daily. His periorbital swelling improved with this therapy, but as the prednisone was tapered he began experiencing symmetric bilateral proximal muscle weakness and soreness of the upper and lower extremities. This was accompanied by a hyperpigmented rash on the trunk, extensor surface of the arms, and upper thighs with associated soft tissue swelling. Two weeks after the completion of the prednisone taper, he began experiencing dysphagia to solids more than to liquids and suffered several choking episodes. This forced a change in his diet to thick liquids; as a result, by the end of the month he had lost 60 pounds. Given his worsening symptoms, he presented to our outpatient clinic in July 2014. Blood work for autoantibodies including ANA and dsDNA was negative. He was found to have an elevated total CK (1274 IU/L). ESR and CRP were normal. An upper endoscopy did not reveal any structural abnormalities.

**Figure 1:**
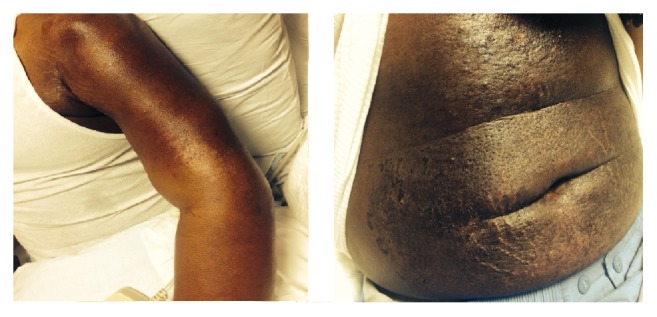
On physical exam, the patient was found to have a patchy, raised, nonulcerative healing rash with hypopigmentation on bilateral arms and forearms and anterior and lateral thighs, a patch of hyperpigmented rash on chest, and a diffuse rash over abdomen.

In August 2014 during a follow-up visit at our clinic he was found to be very weak, barely able to climb stairs or stand up from a seated position. On examination, hyperpigmented pruritic frank macules were noted on the extensor surface of his arms, trunk, and upper thighs in multiple phases of healing (Figure 1). His hands and lips were swollen. Hand grip strength was reduced bilaterally. He had 3/5 strength in the left upper extremity and 4/5 on the right upper extremity. He was not able to raise his arm above his head; muscle bulk was decreased in the upper arm compared to the lower extremity. Hip flexors were 3/5 bilaterally, but quadriceps and hamstrings were 4/5 (Table 1). In this setting he was immediately transferred to a tertiary care facility.

Initial lab work there revealed elevated enzymes with AST of 75 U/L, creatinine kinase level of 1017 IU/L, myoglobin level of 233 ng/mL, LDH level of 386 U/L, ferritin 3151 ng/mL and ESR 33 mm/hr, uric acid 6.8 mg/dL, and being HIV negative (Table 1). Based on the clinical presentation and history, inflammatory myositis was suspected and rheumatology and dermatology were consulted. Blood works including an ANA, anti-Jo-1, anti-Ro, anti-La, anti-RNP/Smith, serum myoglobin, aldolase, CMV/EBV titers, and anti-histone antibodies were all negative.

**Figure 2:**
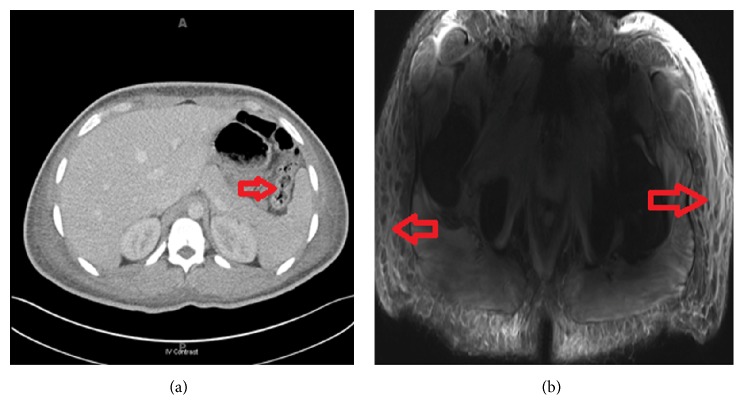
(a) CT of the abdomen showing thickening of the bowel wall (red arrow). (b) MRI showing edema of the soft tissue (red arrows).

He underwent CT scan and MRI of his chest, abdomen, and pelvis. Edema found in the bowel wall on CT (Figure 2(a)) prompted a colonoscopy with biopsy but results were nonspecific. MRI revealed intramuscular and subcutaneous edema (Figure 2(b)), but no occult malignancy. Skin biopsies were obtained from his right extensor arm and anterior chest which revealed a vacuolar interface dermatitis with dermal mucin deposition. A muscle biopsy of his left biceps demonstrated perifascicular atrophy and inflammation consistent with dermatomyositis. For his dysphagia, he was evaluated by a speech-language pathologist and underwent a cine esophagram which showed severe swallowing weakness with aspiration; a PEG tube was subsequently placed. He then began therapy with IVIG and pulse-dose pulse steroids with an eventual taper to 60 mg prednisone. Following IVIG treatment his hemoglobin dropped to 6.9 mg/dL with an increased total bilirubin and LDH suggestive of intravascular hemolysis. Fecal occult blood test was negative and upper-GI bleed was not suspected leading to a glucose-6-phosphate dehydrogenase (G6PD) assay that confirmed very low activity of the enzyme. Thus he was diagnosed with G6PD deficiency and appropriate medication adjustments were made. Hemolysis was thought to be the result of stress from hospitalization. After 1 unit PRBC transfusion his hemoglobin remained stable for the course of the admission and he did not require any more transfusions. He demonstrated clinical improvement over the ensuing days, at which time his antibody testing for NXP-2 antibodies came back positive. NXP-2 antibodies were detected by the Oklahoma Myositis Research Foundation Panel.

## 3. Discussion

Dermatomyositis is a systemic disease that can be difficult to diagnose in its early stages [5]. The presence of myositis-specific autoantibodies allows for a more confident diagnosis, better phenotype classification, and potentially more targeted treatments. Herein we present a patient with NXP-2 positive DM with dysphagia, unusual bowel wall edema, and diffuse soft tissue and muscle inflammation responsive to IVIG and corticosteroids.

Anti-NXP-2 antibodies were originally described in a subset of patients with juvenile DM and were associated with severe muscle weakness, polyarthritis, joint contractures, and intestinal vasculitis [7]. Anti-NXP-2 is also strongly associated with malignancy [8]. Until recently, there has been limited literature regarding its association in adult DM.

A recent study showed 1.6% of adult Japanese patients with inflammatory myopathy had anti-NXP-2 antibodies [9]. Anti-NXP-2 antibodies were found to be the most prevalent specificity in an Italian cohort followed by other known MSAs, such as anti-Jo-1 and Mi-2, and it was found to be similar to two juvenile DM studies performed in Argentina, the United Kingdom, and Ireland [7].

Two recently published studies have demonstrated that NXP-2 autoantibodies are associated with dysphagia and soft tissue/peripheral edema. Rogers et al. observed that in 20 patients with antibodies to NXP-2 dysphagia and peripheral edema were present in 74% and 35%, respectively [10]. In another study by Albayda et al., dysphagia and subcutaneous edema were present in 62% and 36% of anti-NXP-2 positive adult dermatomyositis patients [11]. In both studies, patients negative for NXP-2 autoantibodies had significantly less dysphagia and peripheral edema.

We describe a case of NXP-2 positive DM in a 21-year-old African American man with gastrointestinal involvement and soft tissue edema. This adds to the growing body of evidence that NXP-2 antibodies may reflect a unique phenotype of DM.

## Figures and Tables

**Table 1:** A summary of the significant lab results, immunologic test results, and results from the detailed motor strength test.

Lab results	
AST	75 U/L (0–35 U/L)
CK	1017 IU/L (30–170 U/L)
Myoglobin, serum	233 ng/mL (0–85 ng/mL)
LDH	386 U/L (60–100 U/L)
Ferritin	3151 ng/mL (15–200 ng/mL)
ESR	33 mm/hr (0–15 mm/hr)
Uric acid	6.8 mg/dL (3.7–8.0 mg/dL)
HIV	−ve

Immunologic tests	

ANA	−ve
Anti-Jo	−ve
Anti-Ro	−ve
Anti-LA	−ve
Anti-RNP	−ve
Anti-Smith	−ve
Anti-histone	−ve
CMV/EBV	−ve

Motor strength	

Neck flexion	2/5
Deltoids	3/5 bilaterally
Biceps	4/5 bilaterally
Triceps	4+/5 bilaterally
Wrist flexion	4+/5 on L, 4/5 on R
Wrist extension	4+/5 on L, 4/5 on R
Hand grip	4+/5 bilaterally
Hip extension and flexion	4/5 bilaterally
Knee extension and flexion	4+/5 bilaterally
Ankle flexion and dorsiflexion	5/5 bilaterally

## References

[1] Callen J. P. (2000). Dermatomyositis. *Lancet*.

[2] Callen J. P., Wortmann R. L. (2006). Dermatomyositis. *Clinics in Dermatology*.

[3] Olazagasti J. M., Baez P. J., Wetter D. A., Ernste F. C. (2015). Cancer Risk in Dermatomyositis: A Meta-Analysis of Cohort Studies. *American Journal of Clinical Dermatology*.

[4] Bohan A., Peter J. B. (1975). Polymyositis and dermatomyositis II. *The New England Journal of Medicine*.

[5] Bohan A., Peter J. B. (1975). Polymyositis and dermatomyositis. *The New England Journal of Medicine*.

[6] Zong M., Lundberg I. E. (2011). Pathogenesis, classification and treatment of inflammatory myopathies. *Nature Reviews Rheumatology*.

[7] Ceribelli A., Fredi M., Taraborelli M. (2012). Anti-MJ/NXP-2 autoantibody specificity in a cohort of adult Italian patients with polymyositis/dermatomyositis. *Arthritis Research and Therapy*.

[8] Fiorentino D. F., Chung L. S., Christopher-Stine L. (2013). Most patients with cancer-associated dermatomyositis have antibodies to nuclear matrix protein NXP-2 or transcription intermediary factor 1*γ*. *Arthritis and Rheumatism*.

[9] Ichimura Y., Matsushita T., Hamaguchi Y. (2012). Anti-NXP2 autoantibodies in adult patients with idiopathic inflammatory myopathies: Possible association with malignancy. *Annals of the Rheumatic Diseases*.

[10] Rogers A., Chung L., Li S., Casciola-Rosen L., Fiorentino D. F. (2017). The cutaneous and systemic findings associated with nuclear matrix protein-2 antibodies in adult dermatomyositis patients. *Arthritis Care & Research*.

[11] Albayda J., Pinal-Fernandez I., Huang W. (2017). Dermatomyositis patients with anti-nuclear matrix protein-2 autoantibodies have more edema, more severe muscle disease, and increased malignancy risk. *Arthritis care & research*.

