# Corrigendum to “NXP-2 Positive Dermatomyositis: A Unique Clinical Presentation”

**DOI:** 10.1155/2018/1041738

**Published:** 2018-09-09

**Authors:** Zeeshan Butt, Leeza Patel, Manash K. Das, Christopher A. Mecoli, Alim Ramji

**Affiliations:** ^1^Internal Medicine Residency Program, Prince George's Hospital Center, 3001 Hospital Dr, Cheverly, MD 20785, USA; ^2^Department of Rheumatology, University of Arkansas for Medical Sciences, Little Rock, AR, USA; ^3^Division of Rheumatology, Johns Hopkins University School of Medicine, 733 N Broadway, Baltimore, MD 21205, USA

In the article titled “NXP-2 Positive Dermatomyositis: A Unique Clinical Presentation” [1], an acknowledgment should be added as follows:

CAM was supported by the National Institutes of Health/National Institute of Arthritis and Musculoskeletal and Skin Diseases Grant T32AR048522.

Additionally, there was an error in the third affiliation. The revised affiliations are shown above.
